# Integrating epigenetic, genetic, and phenotypic data to uncover gene-region associations with triglycerides in the GOLDN study

**DOI:** 10.1186/s12919-018-0142-9

**Published:** 2018-09-17

**Authors:** Razvan G. Romanescu, Osvaldo Espin-Garcia, Jin Ma, Shelley B. Bull

**Affiliations:** 1 Lunenfeld-Tanenbaum Research Institute, Sinai Health System, 600 University Ave, Toronto, ON M5G 1X5 Canada; 2 Dalla Lana School of Public Health, University of Toronto, 155 College St, Toronto, ON M5T 3M7 Canada

## Abstract

**Background:**

There has been significant interest in investigating genome-wide and epigenome-wide associations with lipids. Testing at the gene or region level may improve power in such studies.

**Methods:**

We analyze chromosome 11 cytosine-phosphate-guanine (CpG) methylation levels and single-nucleotide polymorphism (SNP) genotypes from the original Genetics of Lipid Lowering Drugs and Diet Network (GOLDN) study, aiming to explore the association between triglyceride levels and genetic/epigenetic factors. We apply region-based tests of association to methylation and genotype data, in turn, which seek to increase power by reducing the dimension of the gene-region variables. We also investigate whether integrating 2 omics data sets (methylation and genotype) into the triglyceride association analysis helps or hinders detection of candidate gene regions.

**Results:**

Gene-region testing identified 1 CpG region that had been previously reported in the GOLDN study data and another 2 gene regions that are also associated with triglyceride levels. Testing on the combined genetic and epigenetic data detected the same genes as using epigenetic or genetic data alone.

**Conclusions:**

Region-based testing can uncover additional association signals beyond those detected using single-variant testing.

## Background

Many authors have called for greater use of gene-based approaches to detect candidate regions at the genome-wide discovery stage, raising concerns that exclusive marginal single-variable testing may miss more complex associations. For example, Yoo et al. [1] report that region tests can be more sensitive to genetic architectures with multiple causal components, and find that reduced-dimension test statistics, such as that proposed by Gauderman et al. [2], can improve power compared to tests in full multivariable regressions. To some extent, this argument also applies to genome-wide epigenetic studies, but conclusive evidence is lacking for it. Specification of the constituent variables for a gene region, however, is a major challenge in implementation for both genetic and epigenetic gene-region modeling, and is critical for integration of the 2 data sources when the molecular technology platforms differ.

In their investigation of epigenome-wide association of fasting blood lipids in the Genetics of Lipid Lowering Drugs and Diet Network (GOLDN) study, Irvin et al. [3] model the percentage methylation separately at each individual cytosine-phosphate-guanine (CpG) site as a function of triglyceride levels. They report genome-wide significant associations of 4 CpG sites in intron 1 of the *CPT1A* gene located on chromosome 11. In this article, we apply gene-region association methods to the original chromosome 11 epigenetic data from the GOLDN study [3], supplemented with chromosome 11 genome-wide association study single-nucleotide polymorphism (SNP) data available in a common subset of individuals. Our aims are to explore the association between baseline triglyceride (TG) levels and genetic/epigenetic factors using gene-region analysis methods, and to investigate 1 approach to integration of 2 omics data types (SNP genotype and CpG methylation) by comparing the integrated approach with separate analyses.

## Methods

### Data

We take the phenotype to be log-transformed triglyceride (lnTG) using averaged TG measurements before treatment with fenofibrate. To investigate genetic association with the phenotype, we convert the Affymetrix platform SNP genotypes to allele counts coded as 0, 1, or 2. In total, we consider 36,796 SNPs on chromosome 11. The methylation data for the same chromosome consists of 28,285 CpG sites in total. The number of participants from the original study with sufficiently complete epigenetic data are 995. Of the 995 participants, 717 had genotype information as well.

### Specification of gene regions

Sets of CpG sites and SNPs corresponding to each gene region were obtained using the GENCODE [4] annotation file bundled with LocusZoom standalone software [5], and expanding each genetic region by 20 kb before the start, and after the end, of the annotated base-pair positions. This was done to include any possibly related functional SNPs or CpG sites from each gene neighborhood. In all, we defined 2621 gene regions on chromosome 11. The number of component variables per gene region ranged from 2 to 544 for SNPs and from 2 to 372 for CpG sites. For computational reasons, we excluded 6 genetic regions (*Metazoa_SRP, SNORA1, SNORA7, U3, Y_RNA, snoU13*) that had more than 2000 CpG or SNPs from the subsequent gene-region regression analysis.

### Single CpG epigenetic association

To investigate association between TGs and methylation on chromosome 11, we regress percentage methylation on lnTG measurements as in Irvin et al. [3], and include age, study site, sex, and cell purity as fixed effects, and family as a random effect. TG values are first averaged over the measurements pretreatment (at most 2 per participant), as this yields the most complete data set (995 cases). Cell purity variables estimated as the top 4 principal components of the methylation data, are included as fixed effects. The model reads (in R notation):

1 $$ \mathrm{CpG}\sim \ln \left(\mathrm{TG}\right)+\mathrm{age}+\mathrm{center}+\mathrm{sex}+ PC{1}^G+ PC{2}^G+ PC{3}^G+ PC{4}^G $$

with a random effect for GPEDID, the family ID from the pedigree file, used to account for familial correlation. Here, the superscript *G* indicates that the principal component (*PC*) for cell purity is computed globally for chromosome 11. We note 2 differences from the original GOLDN study. First, the chromosome 11 methylation data we use to calculate cell purity *PCs*) has 28,285 CpG sites, whereas in Irvin et al. [3] the same procedure was based on 461,281 CpG sites from the whole genome (after quality control). Second, model (1) assumes a common correlation among members of the same family, whereas the original analysis used the kinship coefficient to define the correlation of random effects. Our approach is much faster computationally as it uses the *lmer* function rather than *lmekin* (as in Irvin et al. [3]). We also confirmed the fit using the kinship coefficient, but note that the *p* values obtained using model (1) already match those in the original paper fairly closely.

### Gene-region testing of SNP genetic and CpG epigenetic association

To assess the value of integrating the 2 types of data in detecting gene regions associated with the TG phenotype, we regress lnTG on SNP-derived and CpG-derived predictors. We employ the method of Gauderman et al. [2], which computes the PCs of the regressors, and tests for association between the response (“Y” = lnTG) and the PCs of the “X” variables that explain at least 80% of their total variation. This method takes advantage of the correlation structure within a gene region, and may increase power by reducing the dimensionality of the regressor set, such that more genes achieve significance even if their component CpG sites/SNPs are not detected in marginal regression.

For a given gene, let {${PC}_1^s,{PC}_2^s,\dots, {PC}_k^s$} be the first *k* PCs of the SNP variables associated with that gene, which explain 80% of their variation. Similarly, define {${PC}_1^m,{PC}_2^m,\dots, {PC}_l^m$} as the first *l* PCs of the methylation data. With this reduced data set, we fit the following regression models, including random effects for family:

2 $$ \ln \left(\mathrm{TG}\right)\sim {PC}_1^s+{PC}_2^s+\dots +{PC}_k^s+\mathrm{age}+\mathrm{center}+\mathrm{sex} $$

3 $$ \ln \left(\mathrm{TG}\right)\sim {PC}_1^m+{PC}_2^m+\dots +{PC}_l^m+\mathrm{age}+\mathrm{center}+\mathrm{sex}+ PC{1}^G $$

4 $$ \ln \left(\mathrm{TG}\right)\sim {PC}_1^s+\dots +{PC}_k^s+{PC}_1^m+\dots +{PC}_l^m+\mathrm{age}+\mathrm{center}+\mathrm{sex}+ PC{1}^G $$

We opted to use the first chromosome 11 global *PC*^*G*^ of the methylation data as a measure of cell purity, as we found this produces a CpG test *p* value distribution close to that expected under the null hypothesis. In each of the 3 models [models (2), (3), and (4); fitted via R function *lmer*], a global Wald test is performed on the coefficient estimates $\widehat{\beta}$ of the SNP and/or CpG *PC* terms. Model (4) is designed to assess the combined contribution of the CpG and SNP *PC*s and determine whether the 2 sets of *PC*s make independent contributions. Although we limited testing to chromosome 11, to control the overall Type 1 error level, we specify a genome-wide significance threshold for testing. Counting approximately 20,000 to 30,000 genes (and thus tests) yields a threshold of 2 × 10^− 6^.

### Integration of predictors at the gene-region

To further differentiate the relative contribution of SNPs and CpG sites in model (4), we compute variance inflation factors (VIFs) for each PC as a means to identify multicollinearity among the variables in the joint regression model. High correlation between SNP and CpG components may be undesirable because it can inflate standard error estimates. The VIF in a linear regression is computed as ${VIF}_i={\left(1-{R}_i^2\right)}^{-1}$, where ${R}_i^2$ is obtained by regressing the *i*^th^ predictor on all the other predictors. In our case, as *PC*s in each data set are orthogonal, the *VIF* for a SNP *PC* will be based on its correlation with all the CpG *PC*s, and conversely.

## Results

### Single CpG testing

We reproduced the original study associations [3] for chromosome 11 by fitting model (1) to %methylation for each CpG. Eight CpG sites achieved significance (*p* value < 10^− 7^) with the top 4 sites the same as those found in the GOLDN study in *CPT1A* (Table 1).

**Table 1:** Top epigenetic signals for TGs (Model 1) detected in the GOLDN study data set (*n* = 995)

Mark name	Genes	Position	*p* Value (*lmer*)	*p* Value (*lmekin*)
cg00574958	*CPT1A*	68,607,622	6.52E − 31	1.23e − 35
cg17058475	*CPT1A*	68,607,737	1.61E − 20	1.31e − 21
cg01082498	*CPT1A*	68,608,225	2.21E − 11	2.85e − 12
cg09737197	*CPT1A*	68,607,675	7.30E − 10	9.34e − 10
cg11376147	*SLC43A1*	57,261,198	2.51E − 09	7.53e − 09
cg26989316	*CPT1A*	68,607,257	1.90E − 08	7.56e − 09
cg12556569	*APOA5*	116,664,039	2.25E − 08	4.63e − 10
cg00264754	*LRRC4C*	40,136,810	9.30E − 08	3.39e − 07

### Gene-region testing of CpG’s and SNPs

**Fig. 1:**
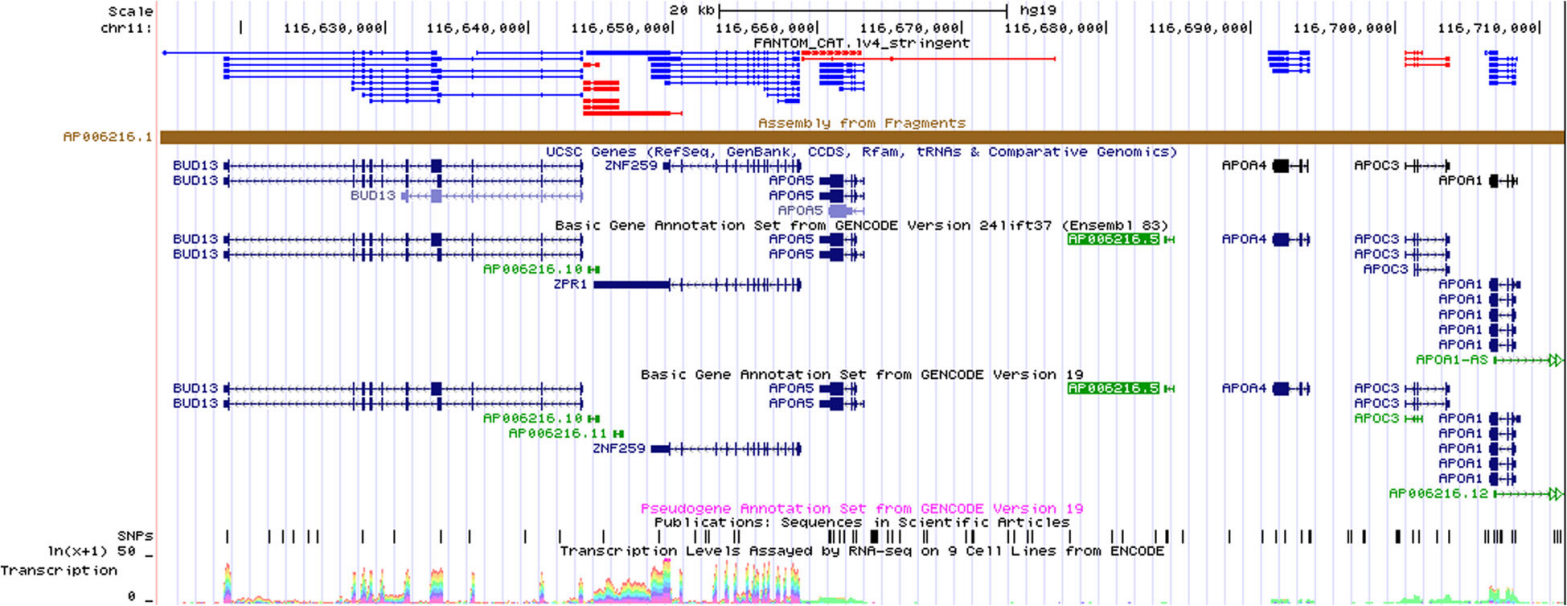
Close-up view of the genetic region containing *BUD13*, *APOA5*, and AP006216.5 (from UCSC Genome Browser)

We fit models (2) to (4) to each gene region in turn, and test the corresponding global association hypotheses for CpG’s and SNPs using generalized Wald tests. We detect gene *CPT1A* using gene-region testing, but in addition we find 2 other genome-wide significant regions: AP006216.5 using methylation data, and *BUD13* using genetic data (Table 2). These gene-regions are in the same neighborhood that also contains *APOA5*, detected in the single CpG analysis reported in Table 1 (Fig. 1).

**Table 2:** Gene-region testing applied to separate epigenetic and genetic regressions (Models 2 and 3; *n* = 717)

Gene	Gene region (BP)		Epigenetic		Genetic	
	Start	End	Degrees of freedom	*p* Value	Degrees of freedom	*p* Value
*CPT1A*	68,522,088	68,611,878	33	3.44e − 14	5	0.436
AP006216.5	116,683,920	116,684,719	7	3.51e − 06	4	0.045
*BUD13*	116,618,886	116,643,704	17	0.538	5	1.48e − 07
*APOA5*	116,660,083	116,663,136	17	1.95e − 04	6	1.47e − 05

The integration of the 2 data types does not seem to improve the overall association signal: testing found the roughly the same gene set to be significant as in the separate epigenetic and genetic analyses, with 3 of the top 4 genes having larger *p* values (see Tables 2 and 3). The examination of pairwise correlations between CpG and SNP *PC*s within gene regions suggests that this can be explained by relationships of higher order CpG PCs with SNP PCs, particularly for *CPT1A* and *APOA5*. *APOA5* was affected in both epigenetic and genetic components. The gene *BUD13* detected in the genetic SNP analysis dropped below the detection threshold after adding CpG data, most likely a consequence of the increase in model degrees of freedom. Remarkably, we observe little Spearman rank correlation between the epigenetic and genetic gene-region *p* values across the 2615 gene regions.

**Table 3:** Gene-region testing applied to integrated epigenetic–genetic regressions (Model 4; *n* = 717)

Gene	BP Start	Epigenetic		Genetic		Epigenetic + Genetic	
	Start	Degrees of freedom	*p* value	Degrees of freedom	*p* Value	Degrees of freedom	*p* Value
*CPT1A*	68,522,088	33	2.47e − 14	5	0.276	38	9.44e − 14
AP006216.5	116,683,920	7	2.62e − 06	4	0.034	11	1.16e − 06
*BUD13*	116,618,886	17	0.711	5	7.13e − 07	22	2.23e − 04
*APOA5*	116,660,083	17	0.088	6	0.064	23	7.19e − 05

**Fig. 2:**
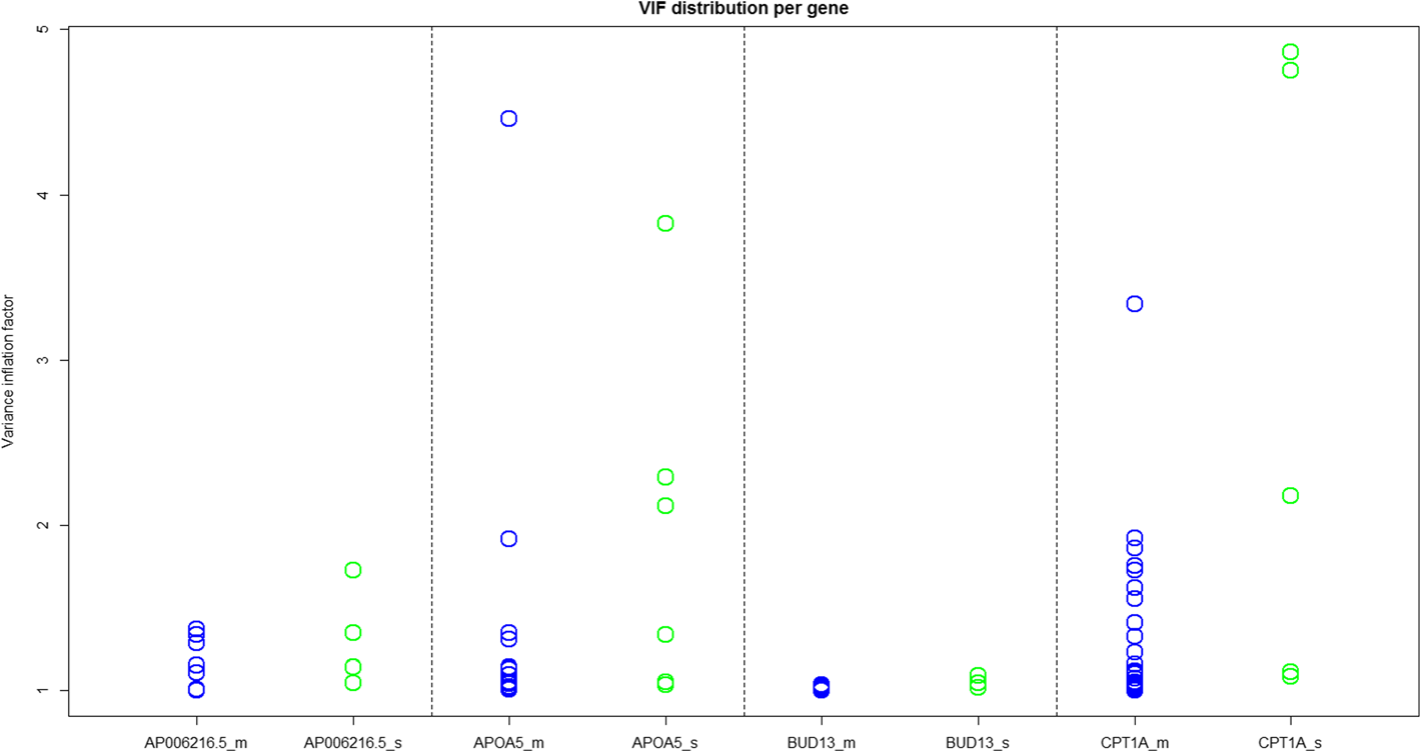
Plots of VIFs for each of the top genes, corresponding to CpG PCs (*blue*) and SNP PCs (*green*)

To address multicollinearity in predictor integration, we fit a reduced model (4) to the 2 genes in Table 3 with high *VIF*s (Fig. 2) by sequentially dropping high *VIF PC*s, until no term remains with a *VIF* larger than 2 (this corresponds to excluding those *PC*s with ≥50% variation explained by the other predictors). This removes SNP *PC1* and *PC2* from the *CPT1A* model, and CpG *PC9* from the *APOA5* model, (with VIFs for all remaining terms below 1.4), but does not improve the overall association signal (*p* values of 1.57e-13 and 9.25e-04 for *CPT1A* and *APOA5*, respectively). For *APOA5*, the most highly collinear CpG *PC* is also one of the strongest predictors of TG, suggesting that *VIF* pruning is not advisable for improving power, but can help clarify variable importance.

## Discussion

**Fig. 3:**
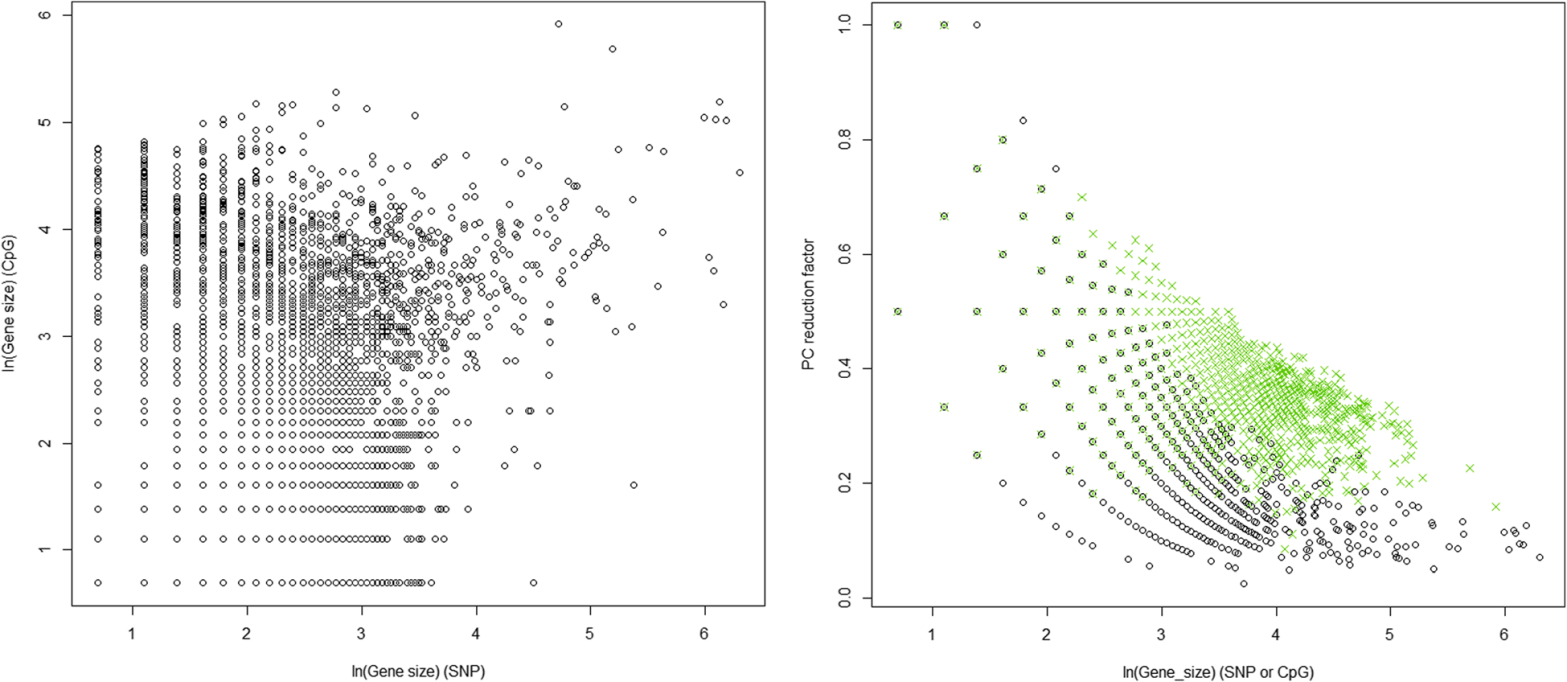
*Left*: Gene sizes (log scale) in SNPs versus CpGs. *Right*: Fraction of dimension reduction achieved by applying Gauderman’s method to SNPs (*black circles*) and CpGs (*green Xs*) on chromosome 11

In this contribution to GAW20, we investigate associations between a lipid phenotype (TG level) and epigenetic (methylation CpG sites) and/or genetic (SNP) markers. As an alternative to single-marker analysis, we apply a gene-region testing method based on multiple regressions of PCs summarizing CpG sites and SNPs in each gene region. The dimension reduction fraction achieved (number of PCs that explain at least 80% of data variability over the number of original variables) was often greater than 50%, with greater data compression for larger genes, and SNP sets showing slightly more dimension-reduction capacity than CpG sites, despite having similar number of original variables (Fig. 3).

In separate and combined epigenetic and genetic regression analyses, we detected genome-wide significant gene-region CpG signals for the *CPT1A* gene reported in the original GOLDN study [3], as well as for 2 other genes. The 2 other gene regions lie within 50 kb of a single significant CpG detected in our single-variable CpG analysis, which suggests that this entire region harbors signals of association with TGs. For the *CPT1A* gene, the epigenetic component clearly leads the results, with no detectable genetic signal. Associations detected with AP006216.5, *BUD13*, and *APOA5*, all located in a different region of chromosome 11, also included epigenetic and/or genetic components. For AP006216.5, the epigenetic component leads the overall association, with an independent nominal genetic component. In contrast, for *BUD13*, the genetic component is the sole contributor. For the *APOA5* gene*,* which is located midway between AP006216.5 and *BUD13*, there is suggestive genome-wide association resulting from both epigenetic and genetic components, which are not independent, and we find evidence for relationships between certain CpG *PC*s and SNP *PC*s. Notably, *APOA5* is a known genetic determinant of TG variation, and recent data points to joint genetic and epigenetic regulation of TG [6].

We attempt to increase power in the combined epigenetic and genetic regression using VIFs to eliminate multicollinearity among predictors. This produces 2 sets of regressors that are approximately orthogonal, facilitating evaluation of independent contributions of SNP- and CpG-based *PC*s, but this approach does not strengthen association signals in the combined regression. We speculate that this may be partly because the *PC*s which are highly correlated between data types are likely to share causal etiology, so excluding them reduces power; and partly because most *PC*s are largely uncorrelated, and the VIF approach does not eliminate these *PC*s. Our recommendation for future studies is that Gauderman’s method works well at the gene level for separate analysis of both genetic and epigenetic data types, and integration of the 2 data sources, with assessment of their intercorrelation, can give further insight.

## Conclusions

Using a gene-region testing approach that effectively reduced predictor dimensionality, we recovered the gene *CPT1A* as having significant association between methylation and TG levels. In addition we identified 2 other genes that were not detected in the single CpG analysis: gene *BUD13*, genetically significant, and region AP006216.5, epigenetically significant. In integration of the genetic and methylation data types when testing for association with TG levels at the gene level, although we found no evidence of improvement in association signal strength over separate analyses, use of a combined model helps clarify the relative contribution of epigenetic and genetic components.
